# Effect of Different Hydrocolloids on the Qualitative Characteristics of Fermented Gluten-Free Quinoa Dough and Bread

**DOI:** 10.3390/foods13091382

**Published:** 2024-04-30

**Authors:** Tiziana Di Renzo, Maria Carmela Trivisonno, Stefania Nazzaro, Anna Reale, Maria Cristina Messia

**Affiliations:** 1 Institute of Food Sciences, National Research Council (ISA-CNR), Via Roma 64, 83100 Avellino, Italy; tiziana.direnzo@isa.cnr.it (T.D.R.); stefania.nazzaro@isa.cnr.it (S.N.); 2 Department of Agricultural, Environmental and Food Sciences (DiAAA), University of Molise, Via De Sanctis, 86100 Campobasso, Italy; mariacarmela.trivisonno@unimol.it (M.C.T.); messia@unimol.it (M.C.M.)

**Keywords:** hydroxypropyl methylcellulose (HPMC), sodium alginate, k-carrageenan, xanthan gum, digital image analysis, yeast fermentation

## Abstract

The aim of this research was to optimize the production process of fermented gluten-free quinoa bread. To this end, the effect of different hydrocolloids on the technological, fermentative, and nutritional properties of quinoa-based gluten-free doughs and breads was evaluated. For this purpose, 3% of four different hydrocolloids (sodium alginate, k-carrageenan, xanthan gum, and hydroxypropyl methylcellulose (HPMC)) were used in gluten-free doughs composed of 50% quinoa flour, 20% rice flour, and 30% potato starch. The rheological and fermentative properties of the doughs were evaluated, as well as the chemical composition, specific volume, crust and crumb color, and alveolar structure profile of gluten-free breads. The results highlighted the differences in dough rheology during mixing and fermentation of the doughs. In particular, HPMC showed a good gas retention (93%) during the fermentation of quinoa dough by registering the highest maximum dough development height (Hm). The gluten-free quinoa breads obtained were characterized by significantly different quality parameters (*p* < 0.05). The use of 3% HPMC resulted in breads with the lowest baking loss, the highest volume, and the most open crumb structure.

## 1. Introduction

The demand for gluten-free products has increased over the past year [1], driven by an increase in the number of people with coeliac disease (CD) and consumers defined as gluten-sensitive. To meet this demand, the volume of gluten-free products is growing rapidly and is fast becoming a global trend.

However, the development of these products represents a major technological challenge, as they are often sensory and nutritionally deficient, leading to consumer dissatisfaction [2].

The most common defects in gluten-free breads are due to inefficient gas expansion and retention during leavening, resulting in reduced bread volume and poor crumb softness [3]. Furthermore, the rheological and fermentative characteristics of many gluten-free breads are very different from those of traditional breads, in which gluten confers unique viscoelastic properties.

Various strategies have been proposed in the scientific literature to improve the overall nutritional value of gluten-free products, to increase their acceptability to consumers, and to find technologically feasible solutions. Most focus on ingredients, additives, enzymes, and other substances (or combinations thereof) that can reproduce the viscoelastic properties of the gluten network and improve the quality characteristics of the final product [4].

Several additives have been used to mimic the viscoelastic properties of gluten and increase the gas-retention ability of the dough [5,6,7]. Among them, several hydrocolloids have been used in the production of various gluten-free bakery products, where they improve the volume, structure, texture, flavor, and overall quality of the final products [8,9]. Different hydrocolloids such as hydroxy-propyl-methylcellulose, carboxymethylcellulose, sodium alginate, pectin, xanthan gum, guar gum, gum arabic, or carrageenans, consisting of a number of water-soluble polysaccharides, have been successfully used as gluten replacers due to their ability to improve dough development and gas retention, increase the texture, and enhance the overall quality and sensory features of gluten-free final products [7,10,11]. Moreover, as also pointed out by Cappelli et al. [4], the literature reports a high degree of variation in hydrocolloids’ functionality. In fact, as ascertained by different authors [9,12,13,14], the positive effects or the limitations of using hydrocolloids in bakery products depend not only on the type and concentration used, but also on the interactions with other dough ingredients and processing conditions. 

Furthermore, many studies have focused on the replacement of wheat flour with flours from pseudocereals such as buckwheat, amaranth, and quinoa, since they have been confirmed to be gluten-free, nutritious, free of allergenic proteins, and also characterized by the presence of numerous bioactive and techno-functional compounds [15,16,17,18,19].

In this research, gluten-free quinoa-based bread was produced, and the effect of four different hydrocolloids (κ-carrageenan, sodium alginate, xanthan gum, and hydroxypropyl methylcellulose) as quality improvers was evaluated. The physicochemical and rheological properties of the bread, color and porosity, dough yield and bread yield percentage, and microstructure were investigated.

## 2. Materials and Methods

### 2.1. Materials and Reagents

Quinoa flour (Sottolestelle srl, San Giovanni Rotondo, Foggia, Italy), rice flour (IPAFOOD srl, Ariano Irpino, Avellino, Italy), potato starch (Paneangeli, Cameo s.p.a., Desenzano del Garda, Brescia, Italy), salt, and sugar (sucrose) were used in the formulation of the gluten-free bread. Four commercially available hydrocolloids were used as improvers: sodium alginate (E401), k-carrageenan (E407), xanthan gum (E415), and hydroxypropyl methylcellulose (HPMC, E464) (Giusto Faravelli S.p.a., Milan, Italy). Fresh liquid yeast (Lievital, Lesaffre Italia s.p.a., Trecasali, Italy) was used as a leavening agent.

The chemicals and reagents used in the study were of analytical grade and were purchased from Sigma-Aldrich Chemistry (Madrid, Spain).

### 2.2. Characteristics of the Flours

Quinoa flour, rice flour, and potato starch were analyzed for moisture (ICC method 109/1) [20], fiber (AACC Method 32.05) [21], ash (ICC method 104/1) [20], total protein (ICC method 105/2) [20], and fat (AACC method 30-20) [21] contents. 

The carbohydrate content was calculated by means of the difference. 

The data reported for all parameters are the averages of three different aliquots of each sample. All results are expressed as g/100 g dry weight (d.w.).

### 2.3. Breadmaking Process

The breads were produced using the direct breadmaking method. A basic bread recipe was used, based on flour weight as described by Turkut et al., [22] with some modifications: 50% quinoa flour (Q) (13.5% moisture basis), 30% potato starch (P), 20% rice flour (R), water (to an optimum consistency of 500 Brabender units), 2% fresh commercial liquid yeast (*Saccharomyces cerevisiae*), 1.75% salt, 1% sugar, and 3% hydrocolloid, as reported in Table 1.

Four doughs were made with the four different hydrocolloids, i.e., k-carrageenan (d-KC), xanthan gum (d-XG), sodium alginate (d-SA), and hydroxypropyl methylcellulose (d-HPMC)). In addition, a control dough (Control) was made by mixing all the ingredients of the recipe without any hydrocolloid (Table 1).

The dough was kneaded in a mixer for 20 min, then rested in a proofer for about 10 min, followed by a further 10 min of kneading. The doughs were placed in molds and, after leavening for about 3 h at 28 °C and 80% relative humidity (RH), the doughs were baked in a deck oven at 180 °C for 40 min.

The dough was prepared in duplicate, and five loaves were produced in each batch. This resulted in two batches of five loaves each. After baking, the loaves were removed from the pans and cooled at room temperature for 120 min before analysis.

### 2.4. Dough Rheological Properties

The effect of the different hydrocolloids on the dough rheology was determined using a farinograph (Brabender, Duisburg, Germany), according to the AACC Method (2000) [21]. The following parameters were determined: water absorption, percentage of water required to achieve a dough consistency of 500 BU (Brabender Units), dough development time (DDT, time to reach maximum consistency in minutes), stability (time during which the dough consistency remains at 500 BU), degree of softening (DS, difference in consistency between the height at peak and to that 10 min later in FU), and farinograph quality number (FQN).

The dough rheology during fermentation was determined using a rheofermentometer (Chopin rheofermentometer F3, Tripette & Renaud, Villeneuve-la-Garenne, France). The procedure for using the rheofermentometer was adapted from that described by Zhu et al. [23]. In detail, each piece of inoculated dough (315 g) was placed in the fermentation vat at 28 °C for 3 h. A piston of 500 g was placed on the dough to measure volume variations during the test. The parameters obtained with the rheofermentometer were Hm (the maximum height in mm of dough development); T1 (the time when the dough reaches the maximum height); Tx (the time at which gas starts to escape from the doughs); Vt (mL of total CO_2_ production); Vr (mL of total CO_2_ retained by the dough); retention coefficient (%).

In our study, the results are expressed as the mean of 3 determinations carried out on 3 different samples for each hydrocolloid used.

### 2.5. Evaluation of Gluten-Free Breads

After baking and cooling, the fresh gluten-free quinoa breads (b-Control, b-KC, b-SA, b-XG, b-HPMC) were removed from the molds, and their main qualitative characteristics were evaluated.

#### 2.5.1. Proximate Composition, Specific Volume, Height, Water Activity, Color, and Baking Loss

The analyses of the proximate composition of quinoa bread samples were carried out. Moisture, ash, and protein contents were determined using ICC methods 110/1, 104/1, and 105/2, respectively [20]. Fat content was determined using the AACC method 30-10.01 [21]. Total starch content was determined using enzymatic assay kits (Megazyme Ltd., Bray, Co. Wicklow, Ireland).

The water activity (a_w_) of gluten-free bread samples was measured using a Rotronic water activity meter (model HP23, HygroPalm, Bassersdorf, Switzerland) at 22 °C.

The specific bulk volume of bread was measured according to the rapeseed displacement following the AACC method 10-05.01 [21].

The specific volume was calculated from the volume/weight ratio and the results are expressed in mL/g.

The baking loss of the bread was determined according to the following formula:

Baking loss % = [(Wbb − Wab)/Wbb] × 100,

where Wbb is the weight of the loaf before baking, and Wab is the weight of the loaf after baking and cooling [24].

As reported by Mariotti et al. [25], the height of the slices (cm) was measured at the centre of the loaf.

Color measurements were carried out using a CR300 colorimeter model (Minolta Italia, S.p.A., Milan, Italy), as previously reported [19]. The color of the crumb and crust of the gluten-free breads was measured using the CIE (Commission Internationale de l‘Eclairage, 1976) L*, a*, b* color system, where L* is the brightness (100 = white; 0 = black), a* is the redness (+, red; −, green), and b* is the yellowness (+, yellow; −, blue).

Additionally, the total color difference (Δ*E*) was calculated using the following equation:

$$\Delta E=\sqrt{{\Delta L}^{*2}+{\Delta a}^{*2}+{\Delta b}^{*2}}$$

where ΔL*, Δa*, and Δb* are the differences in L*, a*, and b* values between the color of the gluten-free breads obtained with the different hydrocolloids (b-KC, b-SA, b-XG, b-HPMC) and the color of the gluten-free control bread (b-Control).

#### 2.5.2. Image Acquisition and Digital Image Analysis

Images of the gluten-free bread slices were taken 24 h after baking. For each gluten-free bread sample, three loaves were sliced transversely using an electric slicer to obtain 15 mm thick slices. They were scanned in color and in black and white using a flatbed scanner (HP ScanJet 8300 Hewlett Packard Co., Palo Alto, CA, USA) with a resolution of 300 dpi and the following settings: 205 highlights 70, shadows 58, and midtones 0.5. The images were saved in TIFF format. The images were analyzed using Software Image-Pro Plus 4.5 (Media Cybernetics, Silver Spring, MD, USA, Windows 98), as described by Reale et al. [26]. Eleven classes were arbitrarily defined, and Supplementary Table S1 shows the minimum and maximum range of alveolar area (mm^2^) for each class.

### 2.6. Statistical Analyses

All determinations were carried out in triplicate. Mean values and standard deviations were calculated. Analysis of variance was used to determine significant differences (*p* ≤ 0.05) between means.

## 3. Results and Discussion

### 3.1. Chemical Composition of the Flours

This study evaluated the possibility of using quinoa flour as the main recipe ingredient for the production of gluten-free bread, as an alternative to commercially available preparations using rice flour, corn starch, tapioca starch, and/or different combinations of legume flours (lentils, chickpeas, etc.). In fact, commercial bread preparations, although supplemented with legume flours, are generally low in protein, carbohydrates, and fiber.

Moreover, the scientific literature reports the benefits of using pseudocereals such as quinoa to improve the nutritional value of foods [27]. Considering the nutritional deficiencies that can occur in coeliac patients due to the malabsorption of some nutrients in the intestine, the inclusion of ingredients with high nutritional value, such as quinoa flour, in the recipe would help to improve the intake of some nutrients by patients.

The chemical composition (g/100 g d.w.) of the flours used in this study is shown in Table 2.

As expected, the protein content of quinoa flour (15.6%) was significantly higher (*p* < 0.05) than that of the other flours (rice flour, potato starch). Furthermore, quinoa flour was characterized by a high amount of fiber (12%), ash (5.59%), and fat (6.1%). Rice flour, on the other hand, was characterized mainly by carbohydrates (89%) and a moderate amount of protein and a low amount of fat, ash, and fiber. Potato flour was characterized mainly by carbohydrates.

As shown in Table 2, the flour blend (QRP) used in the experimentation, constituted by 50% quinoa flour (Q), 30% potato starch (P), and 20% rice flour (R) was balanced in terms of protein (10.1%), fat (3.1%), fiber (6.3%), and carbohydrates (77.5%). Moreover, the fiber content (6.3%) allowed the blend to be claimed to be “high fiber” according to the European Regulation n. 1924/2006.

### 3.2. Rheological Properties of Gluten-Free Doughs

In order to improve the rheological properties of the gluten-free flour mixture (QPR), for potential use in breadmaking, different hydrocolloids were added to the blend. To evaluate the effect of the hydrocolloids on the rheological characteristics of the doughs, farinographic and rheofermentographic analyses were carried out, the results of which are shown in Table 3.

The samples had a water absorption value ranging between 60.4% (d-XG) and 54% (d-SA). The dough development time, defined as the time required for the dough to reach its maximum consistency [28], ranged from 4.5 min (d-KC sample) to 20 min (d-SA). In contrast, the values for dough stability ranged from 1.0 min (d-KC sample) to 13 min (d-HPMC). Both parameters, dough development time and stability, differed greatly between doughs containing different hydrocolloids. The results of farinographic analysis showed that the d-HPMC dough, having a longer stability time (13 min) and a lower degree of softening (5UF), was generally more suitable for the leaving process in breadmaking. HPMC acts as a thickener and stabilizer in bread dough. This effect can be attributed to the high ability of HPMC to absorb more water through hydrogen bonding, resulting in a longer stability time [29,30]. The data obtained from the rheofermentographic analysis showed that the hydrocolloids exhibited different behavior during dough development. The differences in rheofermentographic properties strongly depended on the structure and gas-trapping capacity and were mainly influenced by yeast activity and dough structure. Hm (see Table 3) is extremely relevant to the loaf specific volume of the final products. The dough supplemented with xanthan gum (d-XG) had the lowest Hm value, while the dough supplemented with HPMC had the highest one. The values of Hm showed that HPMC could form a structure more consistently capable of supporting gas retention and dough expansion. Similar results were reported by Liu et al. [31], who found that HPMC supplemented in the potato—wheat dough improved the dough height during the fermentation.

Hydrocolloids also influenced the time of maximum dough development (T1) that was very low for the samples d-XG and high for the other three doughs, reaching the highest value (180 min) in the dough obtained with the addition of HPMC hydrocolloid (d-HPMC).

Table 3 also shows the gas behavior. The time at which gas starts to escape from the doughs Tx (min) varied according to the hydrocolloid used, ranging from 58 min (d-KC) to 81 min (d-HPMC). HPMC showed the highest percentage of gas retained (93%), showing good gas retention during the fermentation process as reported also by other authors [32]. The higher Tx value of d-HPMC doughs compared to d-KC and d-XC doughs could be due to the weaker gelling properties of KC and XC, which limit the dough’s expansion caused by the entrapped gas, resulting in a relatively low Hm value. Moreover, the stiffness of d-KC and d-XG doughs limited the Tx value of the doughs themselves.

### 3.3. Gluten-Free Bread Evaluation

#### Chemical Composition, Water Activity, Loaf Volume, Baking Loss, and Color

Breads produced with the different hydrocolloids and the control breads produced without any hydrocolloid were analyzed for chemical composition (g/100 g d.w.), water activity (a_w_), loaf volume, baking loss, and color. Table 4 shows the values of the chemical composition and water activity (a_w_) of the breads.

The proximate composition of the breads was almost similar, as expected, considering that the starting mix was the same. The breads were characterized for a protein content of approximately 10% d.w., a fat content of about 3% d.w., starch content ranging between 65.6% d.w. (b-SA sample) and 70.2% d.w. of the bread without hydrocolloids (b-control), and ash content comprising between 2.97% d.w. (b-control) and 4% d.w. (b-SA). The moisture content of the breads comprised between 38.7% (b-control) and 50.8% (b-HPMC) (Table 4), highlighting that the hydrocolloids used showed a different ability to retain water in the final product.

The ability of hydrocolloids to retain water could have a positive effect on the shelf-life of the breads. In fact, it has been shown [9] that a higher water content in the dough can extend the shelf life of baked goods and slow down the retrogradation of starch. Staling, the chemical—physical process in which the consistency of the baked product changes and it becomes dry and hard, is one of the most important factors influencing the shelf life and quality of baked goods. The anti-staling effect of hydrocolloids has been extensively studied [32,33,34] and appears to be due to their ability to control and maintain moisture content, stabilize the dough, and influence the structure of the crust [35].

Hydrocolloids such as HPMC, guar gum, xanthan gum, and carboxymethylcellulose have been shown to reduce the gelatinization process of bread, as they limit the mobility of water, which affects the gelatinization process by reducing the enthalpy [36].

Regarding the water activity, the gluten-free bread control (b-control), obtained without the addition of hydrocolloids, had the lowest value (0.960) of water activity content (*p* < 0.05). The highest values of a_w_ (0.990) were recorded for bread crumbs obtained by adding xanthan gum. This result was in agreement with the farinograph data previously exposed, since the dough with xanthan gum had the highest water absorption. The other breads analyzed (b-KC, b-SA and b-HPMC) showed a_w_ values of ~0.97 and were not significantly different from each other. The crumb water activity was slightly increased by the addition of hydrocolloids, in accordance with the results of other authors who found higher a_w_ values in baked goods obtained by using hydrocolloids in the dough recipes [32,37].

The addition of the different hydrocolloids increased both the water activity and the moisture content of the finished bread compared to the control without hydrocolloids.

This result was expected due to the known water retention capacity of hydrocolloids [32].

Table 5 shows the results for the specific volume, baking loss (%), and crumb and crust color of the quinoa-based gluten-free breads.

The specific volumes of the quinoa breads ranged from 1.29 (b-XG) to 2.29 (b-HPMC) mL/g, with significant differences (*p* < 0.05) between the samples. In detail, the control bread sample (b-Control) had values of 1.59 ± 0.03 mL/g. Similar values (1.61 ± 0.05 mL/g) were recorded for the b-KC sample. The highest specific volume value (2.29 ± 0.10 mL/g) was recorded for the bread obtained with the addition of HPMC (b-HPMC), while the bread obtained with the addition of xanthan gum (b-XG), had the lowest volume of 1.29 ± 0.07 mL/g. The b-SA sample had a specific volume of 1.70 ± 0.02 mL/g. Our results are in agreement with other studies reporting that in gluten-free bread, the specific volume shows values between 1.33 mL/g and 2.40 mL/g [38,39]. Hager & Arendt [8] also highlighted that the effect of hydrocolloids on gluten-free model systems varied depending on the raw materials used. In their study, they found that HPMC had a positive linear effect on the volume of teff and maize breads and a negative linear effect on this parameter in rice breads, while the buckwheat bread volume did not change. In our study, HPMC resulted in breads with the highest specific volume as also found by Zhao et al. [40] for gluten-free bread.

**Figure 1:**
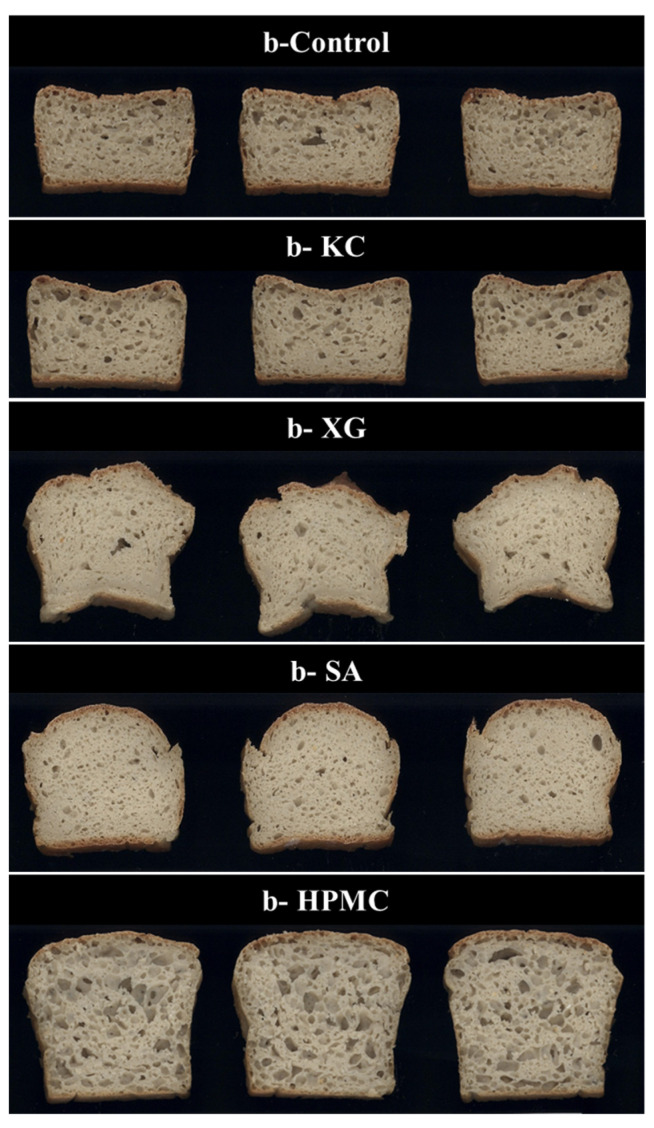
Cross section of fresh gluten-free quinoa-based breads (b) slices obtained using 3% of different hydrocolloids (KC = k-carrageenan, XG = xanthan gum, SA = sodium alginate HPMC = hydroxypropylmethylcellulose).

Figure 1 shows the cross section of fresh slices of gluten-free quinoa bread obtained using the different hydrocolloids.

Looking at the images, visual differences between the different breads were observed. As can be seen from the images, the b-XG and b-SA samples are characterized by the presence of numerous small pores. Larger pores were randomly distributed in the b-control and b-KC samples. The b-HPMC sample, unlike the others, showed uniform alveolation throughout the bread slice.

The values of baking loss are reported in Table 5. The lowest value of baking loss (7.38%) was obtained for breads made by adding HPMC to the dough. Similar values were also recorded for the b-XG sample (7.98%). Significantly different values were recorded for the bread samples obtained via the addition of sodium alginate (10.30%) and k-carrageenan (10.64%), which had values that were not significantly different from the control sample having the highest value of baking loss (11.13%). The baking loss values for quinoa-based breads reported in this study were similar to those found by Franco et al. [41], which were ~9–10.5%. Table 5 also shows the values for the height (cm) of the bread slices. The lowest height (3.83 cm) was recorded for sample b-Control. Similar values were also recorded for sample b-KC (3.93). The samples, b-SA and b-XG, recorded values of 4.90 cm and 5.20 cm, respectively. The highest slice height (6.77 cm) was recorded for sample b-HPMC.

The values for the crust and crumb color of the experimental quinoa breads are shown in Table 5. The color of bread is an important parameter in the commercialization of products and is strongly influenced by the ingredients constituting the formulation and by the baking conditions [22]. The different hydrocolloids used did not significantly affect the crust color of the gluten-free bread compared to the control sample (b-Control). In the crumb, however, significant differences were found for the parameters L*, a*, and b* between the breads obtained by using hydrocolloids and the control sample. In detail, the L* brightness values ranged from 35.8 (b-HPMC) to 58.5 (b-SA). The highest brightness value distinguished the breads obtained by using sodium alginate (b-SA) as the hydrocolloid. Non-significant differences were found between the b-Control, b-KC, and b-XG samples. The a* parameter ranged from 0.3 to 1. No considerable differences in crumb redness were found among the different gluten-free bread samples, except for b-SA, which had lower b* values (0.3) than the other experimental breads. The results highlight the influence of hydrocolloids on the b* values of the crumb. The b* parameter ranged from 18.1 (b-HPMC) to 20.8 (b-XG). Among the different hydrocolloids, slight differences in crumb yellowness characterized the b-KC and b-XG samples. Our results are in agreement with those of other authors, according to whom the use of hydrocolloids in the production of gluten-free bread leads to color changes (L*, a*, b*) mainly in the crumb of the bread [42]. The total color difference (ΔE) showed no significant differences for the crust color between the different gluten-free bread samples. Very slight differences were observed between the crumb colour of the b-HPMC sample and that of the other gluten-free bread samples (b-KC, b-XG and b-SA).

### 3.4. Digital Image Analysis

**Figure 2:**
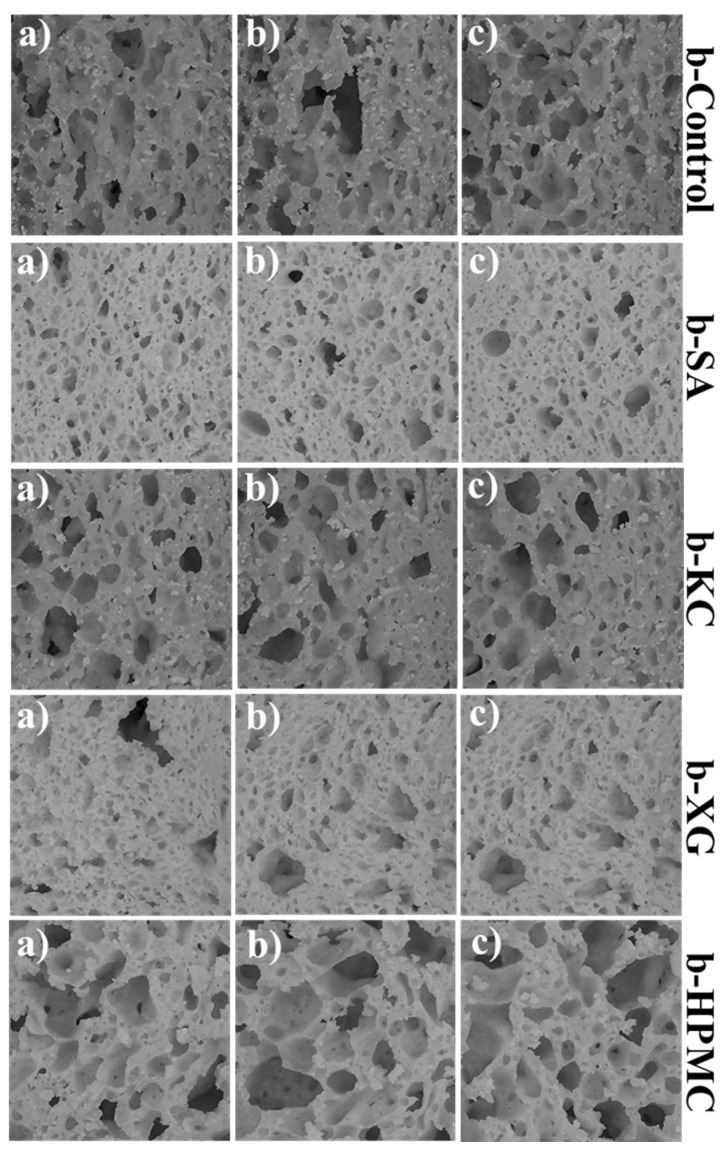
Scanned images of three replicates (**a**–**c**) gluten-free quinoa-based breads (**b**) slices with 3% of different hydrocolloids used for digital image analysis.

Figure 2 shows the scanned images of fifteen slices of fresh gluten-free quinoa-based bread (three from each sample) used for the digital image analysis.

**Figure 3:**
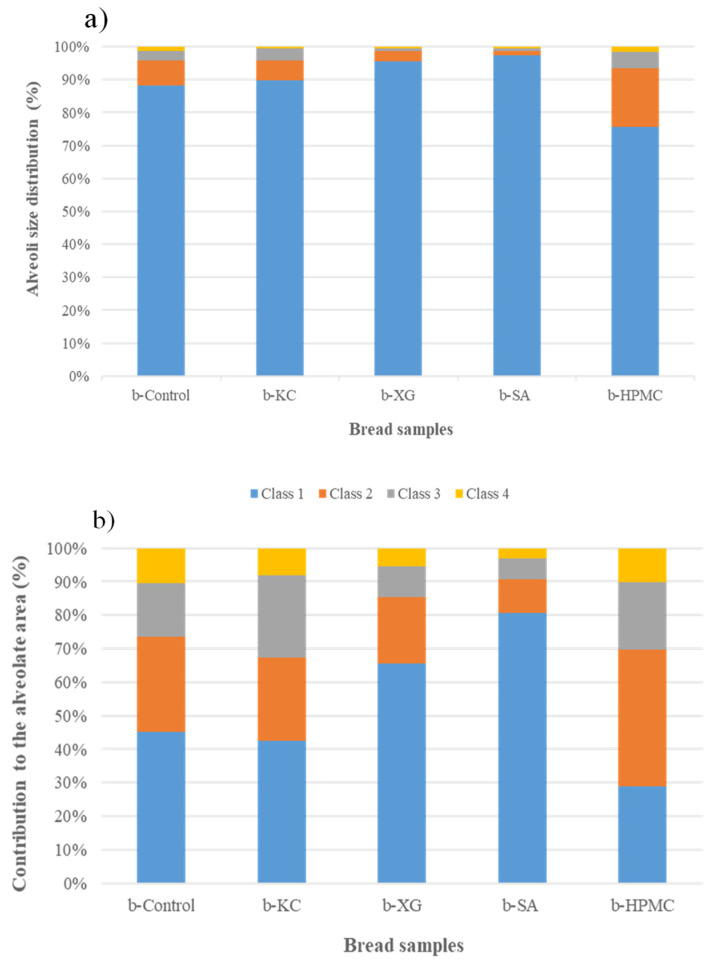
Features of the alveolate surface of the different gluten-free quinoa breads’ crumb: (**a**) alveoli size distribution (%); (**b**) contribution of the different cells’ dimensional classes to the total alveolate area. (class 1: 0.11 < cell size < 3.78 mm^2^; class 2: 3.78 < cell size < 7.47 mm^2^; class 3: 7.47 < cell size < 11.16 mm^2^; class 4: 11.16 < cell size < 14.84 mm^2^).

As reported by Reale et al. [26], the different features of the alveoli (number, size, dimension) in the slice area can be assessed to evaluate the crumb properties of the bread. Figure 3a,b shows the results of the distribution (%) of the size of the crumb alveoli and the contribution (%) of the alveoli of the main classes (1 to 4) to the alveolar area.

The lowest porosity was found in the bread obtained with the addition of sodium alginate (b-SA) and xanthan gum (b-XG). In these samples, the alveoli were found to belong mainly to the first (0.11 < cell size < 3.78 mm^2^) and second (3.78 < cell size < 7.47 mm^2^) classes (Figure 3a).

The bread obtained with the addition of k-carrageenan (b-KC) showed a profile similar to that of the control bread (b-Control). On the other hand, the bread obtained with the addition of HPMC showed a more open crumb structure. In this sample (Figure 3a), a higher percentage of alveoli was detected, also belonging to classes 3 (7.47 < cell size < 11.16 mm^2^) and 4 (11.16 < cell size < 14.84 mm^2^). As reported by Sciarini et al. [43], gluten-free bread has a high cell-wall thickness and a coarse and dense structure due to the inability to incorporate gases during mixing and/or to retain the CO_2_ formed during fermentation due to the lack of a viscoelastic network. Among the hydrocolloids evaluated for the production of quinoa-based bread, HPMC was the one most capable of forming a reversible, heat-set gel network, which can lead to an increase in dough viscosity and stabilization of the boundaries of the expanding gas cells, as already evidenced by other authors [44,45]. In fact, as shown in Figure 3b, in the b-HPMC sample, the alveoli of classes 2, 3, and 4 contributed to the alveolar area of the bread slice, resulting in a bread with a uniform distribution of bubbles and no areas of crumb collapse. Clear areas of crumb collapse near the crust were mainly characteristic of the b-XG and b-SA samples (Figure 1). In addition, the b-XG sample also showed slight deformation of the loaf, as evidenced by the bread slices in Figure 1.

## 4. Conclusions

Hydrocolloids are considered the key ingredients in gluten-free baking, and HPMC is one of the most studied additives. Identifying the technological strategies that can improve the texture, appearance, and fermentative properties of gluten-free bread is a challenge for researchers, given the key role of gluten in the baking process. The results of this study confirmed the possibility of using 50% quinoa flour to develop special leavened breads with good texture characteristics.

Bread made with 50% quinoa flour and containing 3% HPMC had significantly higher volume and better texture characteristics than gluten-free bread made with the other hydrocolloids evaluated. The addition of 3% HPMC had a significant impact on several quality aspects, making it possible to achieve the best technological performance, formulate a gluten-free bread, and develop a baked product with an ameliorated texture profile. Based on the results obtained, we can conclude that quinoa flour, combined with an appropriate hydrocolloid, can be used as a nutritious ingredient in the formulation of yeast-leavened gluten-free bread. However, the concentration of the hydrocolloids may lead to different results, and each hydrocolloid may have its own optimal use concentration. This is probably a limitation of our manuscript, and therefore it will be interesting to evaluate the effect of different concentrations, as well as possible combinations of the hydrocolloids, in further studies.

## Figures and Tables

**Table 1:** Composition of the different quinoa gluten-free dough (d) samples.

Ingredients	Sample Code				
	Control	d-KC	d-XG	d-SA	d-HPMC
Quinoa flour	50	50	50	50	50
Rice flour	20	20	20	20	20
Potato starch	30	30	30	30	30
Salt	1.75	1.75	1.75	1.75	1.75
Sugar	1	1	1	1	1
Yeast	2	2	2	2	2
κ-carrageenan (KC)		3			
Xanthan gum (XG)			3		
Sodium alginate (SA)				3	
Hydroxypropylmethylcellulose (HPMC)					3

**Table 2:** Chemical composition (g/100 g d.w.) of quinoa, rice and potato starch flours used in dough preparation.

Flour Samples	Protein	Fat	Ash	Fiber	Carbohydrates *
**Q**	15.6 ± 0.16 ^a^	6.1 ± 0.10 ^a^	5.59 ± 0.12 ^a^	12.1 ± 1.02 ^a^	60.8 ± 0.50 ^d^
**R**	8.3 ± 0.11 ^c^	0.6 ± 0.05 ^c^	0.71 ± 0.01 ^c^	1.14 ± 0.05 ^c^	89.3 ± 0.21 ^b^
**P**	1.1 ± 0.20 ^d^	0.01 ± 0.01 ^d^	0.35 ± 0.01 ^d^	0.01 ± 0.10 ^d^	98.5 ± 0.21 ^a^
**QRP**	10.1 ± 0.15 ^b^	3.1 ± 0.02 ^b^	3.04 ± 0.12 ^b^	6.3 ± 0.05 ^b^	77.5 ± 0.15 ^c^

Q, quinoa flour, R, rice flour; P, Potato starch; QRP, blend of quinoa (50%), rice (20%), and potato starch (30%) flours. * Calculated by means of the difference. Different superscript letters between means within a column indicate statistically significant differences (*p* < 0.05).

**Table 3:** Rheological and fermentative properties of gluten-free dough (d) samples.

	Sample			
	d-KC	d-SA	d-XG	d-HPMC
**Farinographic index**				
Water absorption (%)	57.5 ^b^	54.0 ^c^	60.4 ^a^	59.2 ^a^
Dough development time DDT(min)	4.5 ^d^	20 ^a^	14.7 ^b^	9.1 ^c^
Stability (min)	1 ^c^	1.5 ^c^	7.3 ^b^	13 ^a^
Degree of softening (FU)	95 ^b^	164 ^a^	74 ^c^	5 ^d^
Farinograph quality number (FQN)	55 ^c^	200 ^a^	147 ^b^	200 ^a^
**Rheofermentographic index**				
Hm (mm)	13.3 ^b^	12.7 ^b^	8.9 ^c^	21.2 ^a^
T1 (min)	177 ^b^	178 ^b^	72 ^c^	180 ^a^
Tx (min)	58 ^b^	79 ^a^	61 ^b^	81 ^a^
Total volume Vt (mL)	1127 ^b^	1051 ^d^	1193 ^a^	1103 ^c^
Retention coefficient Vr (%)	88.1 ^c^	86.9 ^d^	90.7 ^b^	93 ^a^

Means with different letters within the same row are significantly different (*p* < 0.05). Hm (the maximum height in mm of dough development); T1 (the time when the dough reaches the maximum height); Tx (the time at which gas starts to escape from the doughs); Vt (mL of total CO_2_ production); Vr (mL of total CO_2_ retained by the dough).

**Table 4:** Chemical composition and water activity (a_w_) of quinoa gluten-free bread (b) samples.

Samples	Moisture (%)	Protein	Fat	Ash	Starch *	Water Activity (a_w_)
		(% d.w.)	(% d.w.)	(% d.w.)	(% d.w.)	
**b-Control**	38.7 ± 0.26 ^d^	9.8 ± 0.04 ^c^	2.7 ± 0.03 ^a^	2.97 ± 0.078 ^d^	70.2 ± 0.15 ^a^	0.96 ± 0.004 ^c^
**b-KC**	43.8 ± 0.01 ^c^	10.3 ± 0.01 ^a^	2.9 ± 0.14 ^a^	3.35 ± 0.021 ^b^	67.6 ± 0.21 ^b^	0.97 ± 0.002 ^b^
**b-XG**	46.2 ± 0.00 ^b^	9.9 ± 0.03 ^b^	2.7 ± 0.08 ^a^	3.20 ± 0.035 ^c^	67.2 ± 0.13 ^b^	0.99 ± 0.001 ^a^
**b-SA**	46.1 ± 0.10 ^b^	10.1 ± 0.17 ^b^	2.7 ± 0.04 ^a^	4.07 ± 0.064 ^a^	65.6 ± 0.04 ^c^	0.97 ± 0.005 ^b^
**b-HPMC**	50.8 ± 0.34 ^a^	9.7 ± 0.15 ^c^	2.8 ± 0.12 ^a^	3.01 ± 0.035 ^d^	67.9 ± 0.21 ^b^	0.98 ± 0.007 ^b^

* Calculated by means of the difference. Different superscript letters within a column indicate statistically significant differences (*p* < 0.05).

**Table 5:** Specific volume (mL/g), bake loss (%), slice height, crust, and crumb color of the gluten-free quinoa bread (b) samples.

Bread Sample	Specific Volume (mL/g)	Bake Loss (%)	Height (cm)	Color							
				Crust				Crumb			
				L*	a*	b*	ΔE*	L*	a*	b*	ΔE*
**b-Control**	1.59 ± 0.03 ^b^	11.13 ± 0.15 ^b^	3.83 ± 0.06 ^c^	37.5 ± 2.26 ^a^	14.0 ± 0.44 ^a^	28.3 ± 2.10 ^a^	-	48.8 ± 1.36 ^b^	1.0 ± 0.35 ^b^	18.9 ± 0.95 ^a^	-
**b-KC**	1.61 ± 0.05 ^b^	10.64 ± 0.44 ^b^	3.93 ± 0.06 ^c^	40.8 ± 1.00 ^a^	12.0 ± 0.79 ^a^	29.8 ± 1.14 ^a^	4.80 ± 1.82 ^a^	41.3 ± 5.78 ^a^	0.8 ± 0.41 ^b^	20.1 ± 0.64 ^b,a^	8.60 ± 4.97 ^a^
**b-XG**	1.29 ± 0.07 ^a^	7.98 ± 1.11 ^a^	5.20 ± 0.2 ^b^	41.8 ± 2.48 ^a^	13.6 ± 0.35 ^a^	32.5 ± 1.04 ^b^	6.90 ± 3.84 ^a^	45.3 ± 2.92 ^b,a^	0.9 ± 0.33 ^b^	20.8 ± 0.54 ^b,a^	5.00 ± 2.16 ^a^
**b-SA**	1.70 ± 0.02 ^c^	10.30 ± 0.31 ^b^	4.90 ± 0.1 ^b^	39.8 ± 6.50 ^a^	13.0 ± 2.09 ^a^	30.9 ± 2.43 ^a^	7.07 ± 3.88 ^a^	58.5 ± 0.69 ^c^	0.3 ± 0.11 ^a^	18.4 ± 0.33 ^a^	8.80 ± 1.38 ^a,b^
**b-HPMC**	2.29 ± 0.10 ^d^	7.38 ± 0.43 ^a^	6.77 ± 0.06 ^a^	36.0 ± 6.91 ^a^	11.9 ± 0.56 ^a^	29.2 ± 1.02 ^a^	8.30 ± 2.09 ^a^	35.8 ± 3.89 ^a^	0.8 ± 0.33 ^b^	18.1 ± 0.69 ^a^	14.10 ± 4.81 ^b,a^

Mean value of three replicates ± SD. Mean values followed by the same letter are not statistically different (*p* < 0.05).

## Data Availability

The original contributions presented in the study are included in the article/Supplementary Materials, further inquiries can be directed to the corresponding author.

## Supplementary Materials

The following supporting information can be downloaded at: https://www.mdpi.com/article/10.3390/foods13091382/s1, Table S1: Classification of min and max of area (mm^2^) of objects (alveoli) for each class.
