# Supplementary material for: Effect of Different Hydrocolloids on the Qualitative Characteristics of Fermented Gluten-Free Quinoa Dough and Bread
Source: Foods. 2024 Apr 30;13(9):1382. doi: 10.3390/foods13091382 (PMC11083858; doi:10.3390/foods13091382)
Supplement: Supplementary file 1 [file foods-13-01382-s001.zip › foods-2914469-supplementary.pdf]

Supplementary Table S1. Classification of min and max of area (mm^2) of objects (alveoli) for each class.

Class	Range of Area (mm^2)	
	min	max
**1**	0.11	3.78
**2**	3.78	7.47
**3**	7.47	11.16
**4**	11.16	14.84
**5**	14.84	18.53
**6**	18.53	22.21
**7**	22.21	25.90
**8**	25.9	29.58
**9**	29.58	33.27
**10**	33.27	36.95
**11**	36.95	99.99
